# Probabilistic pre-harvest intervals for cyproconazole in green coconut production in Brazil: a Level IV fugacity model with Monte Carlo analysis

**DOI:** 10.1007/s11356-026-38178-w

**Published:** 2026-09-09

**Authors:** Lourival Costa Paraíba

**Affiliations:** Embrapa Meio Ambiente, Rodovia SP-340, Km 127,5, Tanquinho Velho, Jaguariúna, São Paulo, 13918-110 Brazil

**Keywords:** *Cocos nucifera* L., Pre-harvest interval, Fugacity model, Cyproconazole, Monte Carlo simulation, Endotherapy, Pesticide residues

## Abstract

The coconut palm (*Cocos nucifera* L.) fruits continuously, and Brazilian growers apply the triazole fungicide cyproconazole via endotherapy without an established pre-harvest interval, creating a food-safety gap. We coupled a Level IV fugacity model with Gompertz growth functions for the water and pulp compartments to predict cyproconazole residues in green coconut at three doses (1.0, 1.5, 2.0 g palm$^{-1}$) and three frequencies (monthly, bimonthly, quarterly), for fruit harvested at 5–8 months, propagating uncertainty via Monte Carlo simulation (N=2000) and Morris sensitivity screening. The plant-tissue degradation half-life (11.5 days nominal; Pesticide Properties Database) was found to be the dominant determinant of predicted residues relative to the maximum residue limit (0.1 mg kg$^{-1}$; ANVISA 2023; Codex 2023). The 1.0 g dose meets the study’s safety criterion at every frequency and fruit age ($P(C_p>\textrm{MRL})\le 0.024$); risk for 1.5 and 2.0 g is age-dependent, with younger fruit carrying higher exceedance probabilities. A probabilistic harvest-safety matrix is provided as screening-level decision support pending field-residue validation.

## Introduction

The coconut palm (*Cocos nucifera* L.) is cultivated in tropical regions across more than 90 countries, holding significant economic, social, and cultural importance (FAO 2023). In Brazil, production exceeds 2 billion fruits per year, a large proportion of which are consumed fresh as green coconut water and soft pulp (IBGE 2023). Green coconut lacks registered plant protection products for the main fungal diseases affecting the crop, prompting growers to rely on fungicides authorised for other species under agronomic prescription. Among these, the triazole fungicide cyproconazole has been applied via endotherapy to control foliar diseases such as the verrucosis and leaf blight complex (*Camarotella torrendiella* and *Coccostromopsis palmicola*) (Monteiro et al. 2013; Moura et al. 2019).

Beyond its fungicidal action, cyproconazole belongs to a chemical class—the triazoles—with well-documented plant-growth-regulator activity, acting as an inhibitor of gibberellin biosynthesis that shifts resource allocation from vegetative to reproductive growth and can increase flowering and fruit set in treated plants (Desta and Amare 2021). Consistent with this mechanism, Moura et al. (2019) reported that cyproconazole-injected coconut palms produced 57.8% more fruit per year than untreated controls (38.4 additional fruits palm$^{-1}$ year$^{-1}$), an increase substantially larger than would typically be attributed to disease control (improved foliar health) alone. In field practice, this yield-enhancing side effect is anecdotally reported by Brazilian coconut growers and agronomists as producing excessive fruit set on treated bunches, to the point that growers manually thin (abort) young fruits to prevent overloading and yield loss from bunch overcrowding (L.C. Paraíba, personal field observation; not yet systematically documented in the peer-reviewed literature). If confirmed, this dual fungicidal and growth-regulating action would provide an additional, non-disease-related incentive for growers to apply cyproconazole via endotherapy, compounding the food-safety relevance of establishing sound pre-harvest intervals, and is noted here as motivation for future study rather than as an established finding.

The coconut palm exhibits a unique continuous fruiting habit, producing a new bunch approximately every month, with fruits at markedly different developmental stages coexisting on the same plant. When a systemic fungicide is applied via endotherapy, it distributes throughout the vascular system and may reach fruits of all ages simultaneously. This creates an acute challenge for the definition of a pre-harvest interval (PHI), defined as the minimum waiting period between the last pesticide application and harvest that ensures residues do not exceed the maximum residue limit (MRL; 0.1 mg kg$^{-1}$ for cyproconazole on fruits; ANVISA 2023; Codex 2023). No officially established PHI exists for cyproconazole applied via endotherapy to coconut in Brazil or internationally, as the crop is not listed on the fungicide label and no supervised residue trials have been conducted to support regulatory approval. This represents a critical food-safety gap, since green coconut is a major tropical crop produced and consumed at scale but treated as a minor-use crop for pesticide registration purposes.

Pesticide residues in coconut products have been documented in several monitoring studies. Ferreira et al. (2016) detected multiple pesticides including cyproconazole and difenoconazole in coconut water and pulp from different Brazilian regions, with residues remaining below European Union MRLs. Similarly, Suganthi et al. (2021) reported triazole residues in coconut water, kernel, and leaves, finding concentrations below the EU MRL of 0.05 μg g$^{-1}$ for tree nuts and coconut. Pandey et al. (2010) documented organochlorine residues in dried coconut products from Indian markets. Dissipation studies in oil palms—structurally analogous to coconut—have further characterised the fate of triazole fungicides in tropical palm agroecosystems (Maznah et al. 2015).

Mathematical modelling has increasingly been used to determine or refine PHIs without costly field experiments. Fantke and Juraske (2013) reviewed pesticide dissipation half-lives across more than 4500 plant–chemical combinations, establishing the statistical basis for predictive residue modelling. Building on that framework, Fantke et al. (2012, 2013) developed *dynamiCrop*, a multicompartmental plant-uptake tool for high-throughput simulation of pesticide bioaccumulation in harvested crops. Mendez et al. (2018) developed a dynamic multimedia fate model for conventional banana cultivation in Costa Rica, tracking pesticide behaviour across water, soil, air, and plant compartments and finding that, while banana residues remained below regulatory limits, ecosystem exposure via surface water was a persistent concern, illustrating the value of mechanistic environmental fate modelling for tropical plantation crops with continuous production cycles. Modelling residue dissipation in fruit crops has also shown that climate, crop physiology, and application timing interact strongly to determine residue levels at harvest (Hwang and Kim 2025; Horská et al. 2025).

The fugacity concept provides a thermodynamic framework for the partitioning and fate of organic substances across environmental compartments and is classified into four levels of increasing complexity: Level I (equilibrium, closed system), Level II (equilibrium with degradation and advection), Level III (non-equilibrium steady state), and Level IV (fully dynamic, time-dependent) (Mackay and Arnot 2011). Level IV models are particularly suited to biological systems with time-varying compartment sizes, such as growing fruit. Paraíba et al. (2002) extended the Level IV framework to incorporate periodic temperature variation, while Contreras et al. (2008) coupled it with a dispersion-advection equation for pesticide transport in flooded rice fields.

A further methodological challenge is parametric uncertainty. The transport efficiency (η) of endotherapy—the fraction of the applied dose that actually reaches a target fruit—is difficult to measure directly and likely varies with application technique, tree physiology, and environmental conditions. Similarly, the first-order degradation rate constant $k_d$ for cyproconazole in coconut tissues is not available from supervised field trials. Monte Carlo (MC) analysis addresses this limitation by propagating parameter uncertainty through the model, yielding probability distributions of predicted concentrations and explicit exceedance probabilities $P(C > \textrm{MRL})$ (Fantke et al. 2011, 2014). To the best of our knowledge, MC uncertainty analysis has not previously been coupled with a mechanistic fugacity model for PHI estimation in coconut or in any crop treated by endotherapy, where parametric uncertainty in transport efficiency adds a dimension of variability absent from conventional foliar application scenarios.

The present study innovates by coupling a Level IV fugacity model with Gompertz growth functions for coconut water and pulp compartments, capturing the time-varying dilution and lipid-dependent partitioning that govern residue dynamics in developing fruits, and by integrating a full MC uncertainty analysis with 2000 realisations to provide probabilistic rather than purely deterministic PHI estimates. The specific novel contributions of this study, as distinct from the individually pre-existing Level IV fugacity formalism (Mackay and Arnot 2011; Paraíba et al. 2002) and Gompertz growth function (Ahmed 2025), are as follows: (i) the coupling of the periodic Level IV fugacity framework with time-varying Gompertz growth functions (4–5) so that the water and pulp compartments grow rather than remain fixed in size, together with the consequent age-dependent lipid-partitioning formulation (6–8), and (ii) the conservative calibration of the transport efficiency parameter (9) directly against the regulatory MRL, in the absence of measured endotherapy transport data. To our knowledge, this growth-coupled compartmental formulation has not previously been applied to pesticide residue prediction in an endotherapy-treated crop. The specific objectives are as follows: (a) to determine safe PHIs for doses of 1.0, 1.5 and 2.0 g applied at monthly, bimonthly or quarterly intervals for fruits harvested at 5–8 months of age; (b) to quantify the probability of residue exceedance under parametric uncertainty; and (c) to provide graphical and tabular tools that facilitate direct decision support in the field.

The remainder of this article is organised as follows. The “Materials and methods” section describes the Level IV fugacity model, the Gompertz growth and lipid sub-models, the derivation of the transport efficiency and degradation parameters, the Monte Carlo uncertainty analysis, and the Morris global sensitivity and convergence analyses added in this revision. The “Results” section reports the deterministic and probabilistic concentration matrices, the sensitivity screening results, and the Monte Carlo convergence results. The “Discussion” section discusses model behaviour, situates the findings relative to other PHI estimation approaches and independent triazole residue data, addresses regulatory implications and threshold sensitivity, and states the study’s limitations. The “Conclusions” section concludes.

## Materials and methods

### Level IV fugacity model

Fugacity *f* (Pa) is a thermodynamic quantity measuring the escaping tendency of a chemical from a given compartment. The concentration *C* (mol m$^{-3}$) in compartment *i* is related to fugacity by C=Zf, where *Z* (mol m$^{-3}$ Pa$^{-1}$) is the fugacity capacity (Mackay and Arnot 2011). Two compartments within the coconut fruit were considered: pulp (a lipid-containing solid matrix) and coconut water. The Level IV mass balance equations for cyproconazole in water (*w*) and pulp (*p*) are as follows:

1 $$\begin{aligned} \frac{dm_w}{dt} = E_w + D_{wp}(f_p - f_w) - k_d\,m_w \end{aligned}$$

2 $$\begin{aligned} \frac{dm_p}{dt} = E_p + D_{wp}(f_w - f_p) - k_d\,m_p \end{aligned}$$

where $m_w$ and $m_p$ (mg) are the cyproconazole masses; $E_w$ and $E_p$ (mg day$^{-1}$) are input fluxes from endotherapy; $D_{wp}$ (mg day$^{-1}$ Pa$^{-1}$) is the mass-transfer coefficient; $f_w$ and $f_p$ (Pa) are the fugacities; and $k_d$ (day$^{-1}$) is the first-order degradation rate constant, assumed equal in both compartments (PPDB 2023).

The water fugacity capacity is $Z_w = 10^3/(H\,R\,T)$, with $H = 7.19\times 10^{-5}$ Pa m$^{3}$ mol$^{-1}$ (PPDB 2023), R=8.314 J mol$^{-1}$ K$^{-1}$, and T=298.15 K. The pulp fugacity capacity is

3 $$\begin{aligned} Z_p(t) = Z_w\,K_{pw}(t) \end{aligned}$$

where $K_{pw}(t)$ is the time-dependent pulp–water partition coefficient defined in Eq. 7 below.

### Growth functions and lipid sub-model

Because fruit volume changes markedly during development, the classical assumption of constant compartment size was relaxed by coupling the fugacity equations with Gompertz growth functions, a sigmoidal form still widely used to describe biological growth processes (Ahmed 2025):

4 $$\begin{aligned} m_p(t) = m_{p,\max }\exp \!\bigl [-\exp \bigl (-k_g(t - t_m)\bigr )\bigr ] \end{aligned}$$

5 $$\begin{aligned} V_w(t) = V_{w,\max }\exp \!\bigl [-\exp \bigl (-k_g(t - t_m)\bigr )\bigr ] \end{aligned}$$

with $m_{p,\max } = 0.100$ kg, $V_{w,\max } = 0.400$ L, $k_g = 0.035$ day$^{-1}$, and $t_m = 100$ days, consistent with Santana et al. (2011) and Passos et al. (2019).

The lipid fraction of the pulp follows a logistic function calibrated with the data of Aragão et al. (2004) ($R^2 = 0.94$):

6 $$\begin{aligned} f_{\textrm{lip}}(t) = 0.02 + \frac{0.30}{1 + \exp \!\bigl [-0.013(t - 315)\bigr ]} \end{aligned}$$

The pulp–water partition coefficient and the fraction retained in the pulp at the moment of application are, respectively, as follows:

7 $$\begin{aligned} K_{pw}(t) = f_{\textrm{lip}}(t)\cdot 10^{\log K_{OW}} \end{aligned}$$

8 $$\begin{aligned} F_p(t) = \frac{K_{pw}(t)}{1 + K_{pw}(t)} \end{aligned}$$

with $\log K_{OW} = 2.90$ (PPDB 2023). Representative values of $F_p$ are 0.943, 0.977, and 0.980 for fruits of 3, 6, and 8 months, respectively, consistent with the lipid accumulation reported by Kumar and Balakrishna (2009).

### Transport efficiency and degradation

The transport efficiency η is not an empirically measured parameter: no field data currently exist that quantify what fraction of an endotherapy dose reaches a given coconut fruit. In the absence of such data, η was fixed by an explicit conservative (worst-case) premise, stated here as a calibration equation rather than a measured value: a single 2.0 g application is assumed to yield an initial pulp concentration exactly equal to the MRL (0.1 mg kg$^{-1}$) in an 8-month-old fruit, giving the following:

9 $$\begin{aligned} \eta = \frac{0.10 \times 0.100}{2000 \times 0.980} = 5.06\times 10^{-6} \end{aligned}$$

This premise anchors the model to the single most protective reference point available—the regulatory limit itself—rather than to an arbitrary transport fraction, and is deliberately conservative: by construction, the highest single-application dose (2.0 g) applied to the fruit with the greatest retention capacity (8-month-old, highest lipid fraction) is set to graze the MRL exactly. Any lower transport fraction would only reduce predicted residues further. We emphasise, following the same logic applied throughout this revision, that this is a modelling assumption adopted for lack of field data, not a calibrated physical constant; its value in mg kg$^{-1}$ terms is dimensionless (dose fraction reaching the fruit) and it propagates approximately linearly into $C_p$ (10–11), so a two-fold error in η would translate into an approximately two-fold error in all reported concentrations and exceedance probabilities. This linear, transparent propagation is precisely why η was retained as an uncertain (rather than fixed) parameter in the Monte Carlo analysis (the “Monte Carlo uncertainty analysis” section), and why the Morris screening in the “Global sensitivity analysis” section was conducted to formally verify that it is, together with the degradation rate constant, the dominant driver of output uncertainty.

This calibration assumes η is dose-independent, i.e., the fraction of the applied dose reaching a given fruit scales linearly with dose. In practice, endotherapy transport kinetics may be non-linear at higher doses due to saturation of vascular capacity; using the 2.0 g dose as the worst-case upper bound ensures conservative (protective) PHI estimates. Field trials measuring residues at multiple dose levels would allow dose-specific calibration of η and are identified as the single highest-priority experimental need arising from this study (“Limitations and future work”). Because η is calibrated against a regulatory reference value rather than measured experimentally, every pre-harvest interval and exceedance probability reported below remains a model-based estimate whose absolute values require field confirmation.

Degradation was represented by first-order kinetics, using the plant-tissue dissipation half-life reported for cyproconazole in the Pesticide Properties Database: 11.5 days nominal, with a reported range of 3.5–16.0 days across n=5 field crop trials (PPDB 2023). This is the compound’s “on and in plant matrix” dissipation value, distinct from its soil dissipation half-life (129–142 days), which describes a different environmental compartment and is not applicable to residues in living plant tissue. The plant-tissue value is consistent with independent field measurements of triazole dissipation in plant tissue: 3–4 days in grape and 3.0–5.5 days in wheat (Horská et al. 2025), and, most directly relevant given the shared chemical class and the palm fruit matrix, 2.0–2.2 days for the structurally related triazole difenoconazole in date palm fruit across two cultivars (k=0.32–0.35 day$^{-1}$, $R^2 \ge 0.95$; Al-Tawieh et al. 2026). All of these independent values are of the same order of magnitude, confirming that triazole fungicide half-lives in living plant tissue are on the order of days rather than weeks. The nominal half-life of 11.5 days gives $k_d = \ln 2/11.5 = 0.0603$ day$^{-1}$, used as the deterministic (point-estimate) rate constant throughout the “Deterministic concentration curves and PHI matrix” section; the full 3.5–16.0-day range is propagated as an uncertain input in the Monte Carlo analysis (the “Monte Carlo uncertainty analysis” section). The residual pulp mass from application *j* at time $t_{a,j}$, evaluated at harvest $t_c$, is as follows:

10 $$\begin{aligned} M_{p,j}(t_c) = M_0(D)\cdot F_p(t_{a,j})\cdot \exp \!\bigl [-k_d(t_c - t_{a,j})\bigr ] \end{aligned}$$

where $M_0 = D \times 1000 \times \eta$ mg. Because first-order decay is a linear process, the total pulp concentration at harvest is obtained by the superposition principle (Yousef et al. 2024; Fantke et al. 2011)—the net response to multiple applications is the sum of the individual responses:

11 $$\begin{aligned} M_p^{\textrm{total}}(t_c) = \sum _j M_{p,j}(t_c) \end{aligned}$$

and the pulp concentration is $C_p(t_c) = M_p^{\textrm{total}}(t_c) / m_p(t_c)$ (mg kg$^{-1}$). The harvest is considered safe if $C_p(t_c) \le 0.1$ mg kg$^{-1}$.

**Fig. 1:**
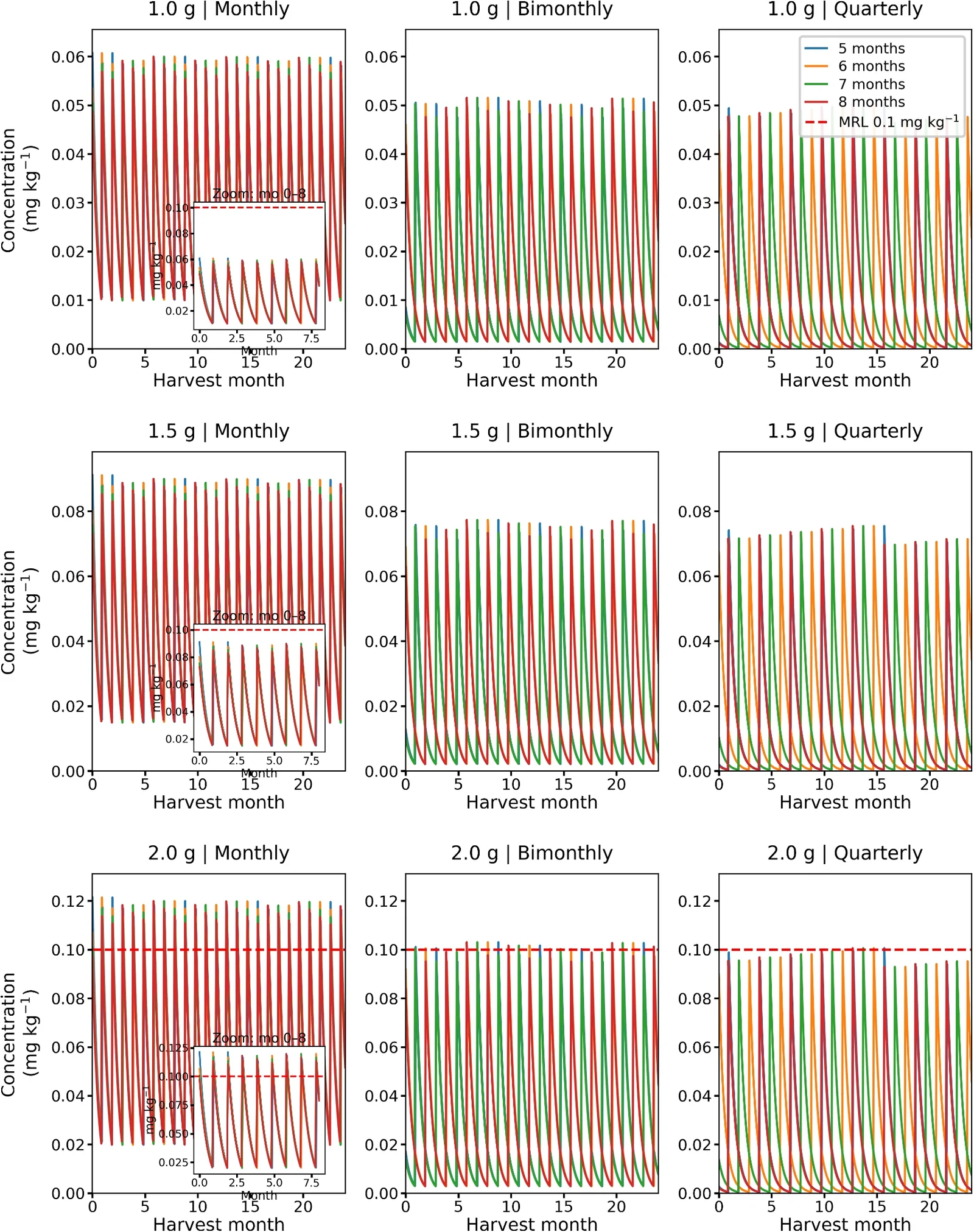
Composite panel of deterministic cyproconazole concentration curves in green coconut pulp for all nine dose–frequency combinations (3 doses × 3 frequencies). Each curve represents a continuous, ongoing endotherapy programme (not a single application): the horizontal axis is the harvest month, and each point sums the residual contributions of all applications from programme start to that harvest time (11). The resulting sawtooth pattern reflects the ∼10–33 applications accumulated by each frequency over the 24-month window, not an error. Colours denote fruit age at harvest (5, 6, 7, 8 months); the red dashed line marks the MRL (0.1 mg kg$^{-1}$). Rows: dose (1.0, 1.5, 2.0 g); columns: frequency (monthly, bimonthly, quarterly). The plant-tissue half-life used is 11.5 days (PPDB 2023); because this is short relative to the application interval, substantial inter-application decay produces the sawtooth pattern at all three frequencies. Insets (monthly column) zoom into months 0–8, showing rapid convergence to the periodic regime

We emphasise that the application times $t_{a,j}$ in Eqs. 10–11 are generated as a continuous, indefinite endotherapy schedule at the stated frequency, starting at t=0 and continuing uninterrupted up to each evaluated harvest time $t_c$—not as a single isolated application or a short, fixed-length treatment episode. This reflects the agronomic reality described in the “Introduction” section: the coconut palm fruits continuously, so growers who adopt endotherapy for disease control apply it on an ongoing basis to protect successive bunches, rather than as a one-off treatment. Consequently, the number of applications contributing to $M_p^{\textrm{total}}(t_c)$ grows with $t_c$: for example, at the far right of the harvest-month axis used in Fig. 1 and the “Monte Carlo uncertainty analysis” section (∼29–32 months of elapsed programme time, combining the 24-month calendar scan with the 5–8-month fruit age), the monthly schedule has accumulated approximately 30–33 individual applications, the bimonthly schedule 15–17, and the quarterly schedule 10–11—which is why the concentration curves in Fig. 1 show this many peaks rather than the handful that a single treatment episode would produce. This design choice is deliberate and, we believe, appropriate for PHI determination: because pre-harvest intervals are meant to remain valid for the full operational life of a managed orchard, not only for a grower’s first application, the reported “worst harvest month” in Tables 1 and 2 corresponds to the stabilised periodic (sawtooth) regime reached after the initial transient, which is the realistic long-term exposure a consumer would face under sustained commercial use.

**Table 1:** Screening-level pre-harvest intervals (PHI, months) for green coconut treated with cyproconazole by endotherapy — deterministic model, using the plant-tissue degradation half-life (11.5 days; “Transport efficiency and degradation”). PHI = 0: harvest can be performed immediately after the last application. *n*.*r*., not recommended (residues exceed the MRL in at least one harvest month)

Dose (g)	Frequency	Fruit age at harvest			
		5 mo	6 mo	7 mo	8 mo
1.0	Monthly	0	0	0	0
1.0	Bimonthly	0	0	0	0
1.0	Quarterly	0	0	0	0
1.5	Monthly	0	0	0	0
1.5	Bimonthly	0	0	0	0
1.5	Quarterly	0	0	0	0
2.0	Monthly	n.r.	n.r.	n.r.	0
2.0	Bimonthly	0	0	0	0
2.0	Quarterly	0	0	0	0

**Table 2:** Monte Carlo probability of MRL exceedance $P(C_p > \textrm{MRL})$ at the worst harvest month, by dose, frequency, and fruit age (N=2000; $\eta \sim$ LogNormal, CV = 30%; $k_d$ derived from $t_{1/2} \sim$ Triangular(3.5, 11.5, 16.0 days), the PPDB plant-tissue dissipation range for cyproconazole). Safe: P<0.05; moderate risk: 0.05≤P<0.20; not recommended: P≥0.20. The P<0.05 and P<0.20 boundaries are model-based risk-communication criteria adopted in this study, not regulatory limits; the only regulatory value used here is the MRL itself (0.1 mg kg$^{-1}$). Monte Carlo standard error $\textrm{SE}=\sqrt{P(1-P)/N}$ ranges 0.0005–0.0111 across all cells (the “Monte Carlo analysis: uncertainty propagation” section)

Dose (g)	Frequency	Fruit age at harvest			
		5 mo	6 mo	7 mo	8 mo
1.0	Monthly	0.024	0.009	0.006	0.004
1.0	Bimonthly	0.002	0.002	0.000	0.000
1.0	Quarterly	0.001	0.001	0.000	0.000
1.5	Monthly	0.249	0.135	0.092	0.075
1.5	Bimonthly	0.059	0.059	0.027	0.027
1.5	Quarterly	0.052	0.052	0.019	0.019
2.0	Monthly	0.602	0.436	0.359	0.312
2.0	Bimonthly	0.268	0.268	0.173	0.173
2.0	Quarterly	0.239	0.239	0.132	0.132

### Monte Carlo uncertainty analysis

Parametric uncertainty was propagated using Monte Carlo simulation with N=2000 realisations (seed = 42 for the primary run; see the “Monte Carlo convergence analysis” section for a multi-seed convergence analysis). Two parameters were treated as random variables: transport efficiency η and degradation rate constant $k_d$. Their selection as the sole uncertain inputs is not an a priori assumption but is justified formally by the Morris global sensitivity screening across seven candidate parameters described in the “Global sensitivity analysis” section, which confirms that η and $k_d$ dominate output uncertainty by roughly an order of magnitude over octanol–water partitioning and growth-function parameters.

The transport efficiency η was sampled from a log-normal distribution, chosen because η is a strictly positive ratio bounded below by zero, for which the log-normal is the standard default choice in the absence of compound-specific distributional data. The distribution was parameterised with CV = 30%: $\sigma _{\ln } = \sqrt{\ln (1 + 0.09)} = 0.294$ and $\mu _{\ln } = \ln (\eta _{\det }) - \sigma _{\ln }^2/2$, where $\eta _{\det } = 5.06\times 10^{-6}$.

The degradation rate constant $k_d$ was sampled by first drawing the plant-tissue half-life $t_{1/2}$ from a triangular distribution directly parameterised on the PPDB field-crop range for cyproconazole (minimum 3.5 d, mode 11.5 d, maximum 16.0 d, n=5; PPDB 2023) and then converting via $k_d = \ln 2 / t_{1/2}$. Parameterising directly on the reported empirical range, rather than assuming a symmetric CV around a point estimate, avoids introducing an arbitrary variance structure where the underlying field data already define asymmetric bounds; a triangular distribution was chosen over a uniform one because it weights the reported nominal (modal) value of 11.5 d more heavily than the extremes, consistent with the original PPDB characterisation of that value as the representative central estimate.

The two parameters were sampled independently. We acknowledge that transport and degradation are both plausibly temperature-dependent processes and could therefore be physically correlated (e.g. warmer conditions accelerating both vascular flow and enzymatic/photochemical degradation). No published data currently quantify this correlation for cyproconazole in coconut endotherapy, so independence was retained as the simplest defensible assumption; we note, however, that positive correlation between η and $k_d$ would tend to be self-compensating for $C_p$ (higher transport partially offset by faster decay), whereas negative correlation would be conservative in the direction already adopted for η. Quantifying this correlation is identified as a future research need (“Limitations and future work”).

For each of the 2000 realisations, the full model (1–11) was solved for all combinations of dose (1.0, 1.5, 2.0 g), application frequency (monthly, bimonthly, quarterly), fruit age at harvest (5, 6, 7, 8 months), and harvest calendar month (0–24)—the last representing the point, within an ongoing and indefinite endotherapy programme (“Transport efficiency and degradation”), at which harvest is evaluated, rather than 24 separate one-off treatment episodes. The output for each scenario is a distribution of 2000 predicted concentrations, from which the mean, standard deviation, percentiles $p_{50}$, $p_{90}$, and $p_{95}$, and the probability of exceedance $P(C_p > \textrm{MRL})$ were computed, together with its Monte Carlo standard error, $\textrm{SE} = \sqrt{P(1-P)/N}$. A scenario was classified as safe if P<0.05, as moderate risk if 0.05≤P<0.20, and as not recommended if P≥0.20. These boundaries are model-based risk-communication criteria adopted in this study, not regulatory limits: they follow no single binding regulatory standard—Brazilian and Codex frameworks for registered uses typically require the $p_{95}$ residue (or a comparable high percentile) to remain below the MRL rather than defining explicit exceedance-probability bands—and are used here as a transparent, literature-consistent risk-communication convention for an unregistered (minor) use, analogous to probabilistic bands used elsewhere in pesticide exposure assessment (Fantke et al. 2011, 2014). To test the sensitivity of the classification to this choice, we recomputed the “safe” boundary at P<0.01 instead of P<0.05: five of the 36 dose–frequency–age combinations that were “safe” under the P<0.05 criterion (the 1.0 g monthly scenario for 5-month fruit and the 1.5 g bimonthly and quarterly scenarios for 7–8–month fruit, *P* = 0.019–0.027) would instead be reclassified as “moderate risk” under the stricter P<0.01 criterion, while no scenario changes classification at the upper (0.20) boundary. We report both the P<0.05 matrix (Table 2) and this sensitivity explicitly so that regulators may apply a stricter threshold if warranted. Simulations were implemented in Python 3.12 using NumPy, Matplotlib, Pandas, and SciPy.

### Global sensitivity analysis

To justify restricting the Monte Carlo analysis to η and $k_d$ rather than propagating uncertainty across every model parameter, a Morris elementary-effects screening (Campolongo et al. 2007) was performed across seven candidate parameters: transport efficiency (η), degradation rate constant ($k_d$), octanol–water partition coefficient ($\log K_{OW}$), the asymptotic lipid fraction and inflection midpoint of the logistic lipid sub-model (6), and the Gompertz growth-rate constant ($k_g$) and inflection time ($t_m$, Eqs. 4–5). Each parameter was assigned a physically plausible range centred on its deterministic value (e.g., $k_d$ spanning the same 3.5–16.0-day PPDB half-life range used in the Monte Carlo analysis; $\log K_{OW}$ spanning 2.5–3.3; growth and lipid parameters spanning ±20–35% of their calibrated values). Twenty Morris trajectories (six levels per parameter) were generated, yielding elementary effects $EE_i = [y(x + \Delta e_i) - y(x)]/\Delta$ for each parameter *i*, evaluated on the representative worst-case scenario (1.5 g, monthly, 5-month fruit). The mean absolute elementary effect $\mu ^{*}_i$ and its standard deviation $\sigma _i$ were computed across trajectories to rank parameter importance and detect non-linear or interaction effects (Campolongo et al. 2007).

### Monte Carlo convergence analysis

To address the possibility that classification near the 0.05/0.20 decision boundaries could be sensitive to Monte Carlo sampling noise, the exceedance probability $P(C_p>\textrm{MRL})$ for the representative 1.5 g monthly, 5-month-fruit scenario was recomputed at five sample sizes (N= 500, 1000, 2000, 4000, 8000) and five independent seeds (42, 7, 123, 2024, 31415) at each *N*, allowing the empirical seed-to-seed standard deviation of *P* to be compared against the theoretical binomial Monte Carlo standard error, $\textrm{SE}_{\text {theory}} = \sqrt{\bar{P}(1-\bar{P})/N}$.

## Results

### Lipid sub-model and growth calibration

The logistic lipid fraction model (6) reproduced the experimental values of Aragão et al. (2004) with a mean absolute error of 2.4 percentage points for ages 6–11 months ($R^2 = 0.94$). The Gompertz growth functions yielded a water volume of approximately 400 mL and a pulp mass of approximately 100 g at 8 months, consistent with Passos et al. (2019) and Santana et al. (2011). The fraction retained in the pulp $F_p$ increased from 0.943 at 3 months to 0.980 at 8 months, reflecting the progressive lipid accumulation that enhances sequestration of cyproconazole ($\log K_{OW} = 2.90$) in the pulp matrix.

**Table 3:** Morris elementary-effects screening results (R=20 trajectories, 6 levels), ranked by mean absolute elementary effect $\mu ^{*}$, for the representative 1.5 g monthly, 5-month-fruit scenario

Rank	Parameter	$\mu ^{*}$	σ	Role
1	Transport efficiency (η)	0.0874	0.0317	Uncertain (MC)
2	Degradation rate constant ($k_d$)	0.0685	0.0315	Uncertain (MC)
3	$\log K_{OW}$	0.0014	0.0009	Fixed
4	Lipid logistic midpoint	0.0007	0.0008	Fixed
5	Lipid plateau fraction	0.0004	0.0004	Fixed
6	Gompertz growth-rate constant ($k_g$)	0.0004	0.0004	Fixed
7	Gompertz inflection time ($t_m$)	0.0001	0.0001	Fixed

### Deterministic concentration curves and PHI matrix

Figure 1 presents the 3×3 composite panel of deterministic concentration curves for all nine dose–frequency combinations, computed with the degradation rate constant $k_d = \ln 2/11.5\,\textrm{d}^{-1} = 0.0603$ day$^{-1}$ (“Transport efficiency and degradation”). For the 1.0 g dose, every frequency–age combination remains below the MRL: the peak concentration for monthly-treated 5-month fruit is 0.061 mg kg$^{-1}$. For the 1.5 g dose, all frequencies are likewise below the MRL under the deterministic (point-estimate) model, with the highest peak—0.091 mg kg$^{-1}$, monthly application, 5-month fruit—approaching but not exceeding the limit. For the 2.0 g dose, monthly application is not recommended for fruit harvested at 5–7 months (peak 0.121, 0.107 and 0.101 mg kg$^{-1}$, respectively) but falls below the MRL by 8 months (0.097 mg kg$^{-1}$); bimonthly and quarterly 2.0 g schedules remain below the MRL at every fruit age under the deterministic model. The deterministic PHI matrix is summarised in Table 1. As detailed in the “Monte Carlo analysis: uncertainty propagation” section, the Monte Carlo analysis reveals a materially different picture for several of these deterministically “safe” cells once parameter uncertainty is propagated, underscoring the value of the probabilistic approach over point estimates alone.

We emphasise that Table 1 reflects only the deterministic (point-estimate) model. As the “Monte Carlo analysis: uncertainty propagation” section and Table 2 show, several deterministically “safe” (PHI = 0) cells carry non-trivial probabilities of MRL exceedance once realistic parameter uncertainty is propagated; Table 2, not Table 1, should be used as the primary screening-level decision-support tool.

### Monte Carlo analysis: uncertainty propagation

With N=2000 realisations and the degradation half-life distribution described in the “Transport efficiency and degradation”–“Monte Carlo uncertainty analysis” sections, the Monte Carlo analysis confirms that 1.0 g falls in the safe category at every frequency and fruit age ($P(C_p>\textrm{MRL}) \le 0.024$), that risk becomes age- and frequency-dependent for 1.5 and 2.0 g, and that several deterministically “safe” cells in Table 1 in fact carry moderate or not-recommended exceedance probabilities once parameter uncertainty is propagated. Table 2 reports $P(C_p > \textrm{MRL})$, its Monte Carlo standard error, and the corresponding risk classification at the worst harvest month for each scenario.

For the 1.0 g dose, $P(C_p>\textrm{MRL})$ remained below 0.025 at every frequency and fruit age, and below 0.01 for bimonthly and quarterly schedules specifically ($\textrm{SE} \le 0.003$ in all cases), a margin robust to the choice of criterion (the “Monte Carlo uncertainty analysis” section). For the 1.5 g dose, risk is markedly age-dependent: monthly application is not recommended for 5-month fruit (P=0.249) but falls to moderate risk from 6 months onward (P=0.075–0.135); bimonthly and quarterly schedules fall in the moderate-risk band for 5–6-month fruit (P=0.052–0.059) and become safe by 7–8 months (P=0.019–0.027). For the 2.0 g dose, monthly application is not recommended at every fruit age (P=0.312–0.602); bimonthly and quarterly schedules are not recommended for 5–6-month fruit (P=0.239–0.268) but fall to moderate risk by 7–8 months (P=0.132–0.173). Across all 36 dose–frequency–age combinations, the Monte Carlo standard error ranged from 0.0005 to 0.0111, confirming that the classification is not an artefact of sampling noise except close to the exact 0.05/0.20 boundaries themselves (see the threshold-sensitivity analysis in the “Monte Carlo uncertainty analysis” section and the convergence analysis in the “Monte Carlo convergence” section). The $p_{95}$ concentration exceeded the MRL in every scenario classified as moderate risk or not recommended and remained at or below the MRL in every scenario classified as safe, confirming internal consistency between the exceedance-probability and percentile-based views of the same simulated distributions. We note that, within a given dose and frequency, exceedance probability decreases monotonically with fruit age at harvest (5 months consistently riskier than 8 months): because harvest time in the model is fixed by fruit age plus calendar month, older fruit correspond to a longer elapsed time since the relevant applications, allowing more degradation before harvest.

### Global sensitivity analysis

The Morris screening (described in the “Global sensitivity analysis” subsection of “Materials and methods”; R=20 trajectories, representative scenario: 1.5 g monthly, 5-month fruit) confirms that η ($\mu ^{*}=0.087$) and $k_d$ ($\mu ^{*}=0.068$) are the two most influential parameters, each roughly one order of magnitude more influential than the next-ranked parameter, $\log K_{OW}$ ($\mu ^{*}=0.0014$); the lipid-model midpoint, lipid plateau, growth-rate constant, and growth inflection time followed, all with $\mu ^{*} < 0.001$ (Table 3). This formally supports the choice, made in the “Monte Carlo uncertainty analysis” section, to restrict the Monte Carlo uncertainty propagation to η and $k_d$: the remaining five parameters contribute negligibly to output variance by comparison and their omission does not meaningfully underestimate total uncertainty, confirming that restricting the Monte Carlo analysis to these two parameters is not an unjustified simplification.

### Monte Carlo convergence

The multi-seed convergence analysis (the “Monte Carlo convergence analysis” section; representative scenario: 1.5 g monthly, 5-month fruit, harvest month 5) shows that $P(C_p>\textrm{MRL})$ stabilises as *N* increases: at N=2000 (the sample size used throughout this study), P=0.065±0.007 (mean ± SD across 5 seeds), consistent with the theoretical Monte Carlo standard error of 0.0055 for a probability near this value; at N=8000, P=0.066±0.002. The close agreement between the empirical seed-to-seed spread and the theoretical binomial standard error at every tested *N* (Table 4) indicates that N=2000 was already adequate for stable classification away from the exact 0.05/0.20 decision boundaries, and that the sampling noise contributing to boundary cases in Table 2 is of the order of ±0.005–0.011, consistent with the SE values reported there.

**Table 4:** Convergence of $P(C_p>\textrm{MRL})$ with sample size *N*, across 5 independent seeds (42, 7, 123, 2024, 31415); scenario: 1.5 g monthly, 5-month fruit, harvest month 5

N	Mean *P*	SD across seeds	Theoretical MC-SE
500	0.0616	0.0122	0.0108
1000	0.0644	0.0096	0.0078
2000	0.0650	0.0072	0.0055
4000	0.0661	0.0028	0.0039
8000	0.0662	0.0019	0.0028

## Discussion

### Model behaviour and the superposition principle

The concentration patterns in Fig. 1 arise directly from the superposition principle (11): because first-order decay is a linear process, the total cyproconazole mass in the pulp at any harvest time is the sum of residual contributions from all previous applications (Fantke et al. 2011; Yousef et al. 2024). Ignoring past applications would drastically underestimate concentrations in older fruits, a well-documented shortcoming of single-application PHI models (Fantke and Juraske 2013). This dynamic is especially critical for the coconut palm, whose continuous fruiting habit means that every endotherapy application exposes fruits at all developmental stages simultaneously.

With the 11.5-day plant-tissue half-life, the monthly application interval spans roughly 2.6 half-lives, so each successive dose is substantially degraded before the next is applied. All three application frequencies therefore show a sawtooth accumulation pattern, with the sawtooth amplitude largest—and troughs shallowest relative to peaks—for the monthly schedule. This is the expected qualitative behaviour for first-order decay when the application interval exceeds roughly two half-lives, and it means that growers and regulators can meaningfully use the timing of harvest relative to the last application (not only the schedule and dose) to reduce risk within a given frequency–dose combination; the worst-case harvest month reported in Tables 1 and 2 corresponds to harvesting shortly after an application, before the next inter-application decay interval has elapsed.

A key finding is that the 1.5 g monthly schedule is not recommended for young (5-month) fruit (P=0.249). This results from residue accumulation across successive applications interacting with the high lipid content of mature coconut pulp, which retains lipophilic triazoles more effectively than younger, lipid-poor fruits. This kind of multi-application residue accumulation echoes the broader lesson from dynamic environmental fate modelling of tropical plantation crops such as banana (Mendez et al. 2018): under continuous cultivation cycles with repeated agrochemical inputs, dynamic, multi-application modelling approaches are needed to capture residue behaviour accurately, whereas single-application or steady-state approximations can be misleading. This supports the appropriateness of the dynamic fugacity approach adopted here for tropical plantation crops with multi-age fruit dynamics.

### Comparison with PHI estimation methods in other crops

Mechanistic fugacity models complement, but contrast with, the empirically driven approaches most commonly used in PHI estimation. Hwang and Kim (2025) applied double-exponential decay models to apples, demonstrating that simple first-order kinetics cannot adequately capture biphasic dissipation patterns. Horská et al. (2025) found, for six triazoles in two apple cultivars over 4 years, that dissipation rates varied by an order of magnitude depending on application timing relative to harvest ($t_{1/2}$: 4.0–43.9 days). While empirical approaches require supervised field trials, the fugacity model used here provides a mechanistic alternative parameterisable from physicochemical properties and published lipid growth data, making it especially valuable for minor-use crops where field trial data are absent.

The *dynamiCrop* platform (Fantke et al. 2012, 2013) offers a similar multicompartmental approach validated across numerous crop–pesticide combinations. The Level IV model presented here extends that framework by coupling compartment volumes to Gompertz growth functions, essential for coconut where pulp mass increases sixfold and water volume fourfold during the 5–8-month harvest window. The lipid fraction calibrated from Aragão et al. (2004) rises from approximately 2% in young fruits to approximately 30% in mature ones, shifting $K_{pw}$ from approximately 158 to 251 and substantially increasing the pulp’s capacity to retain cyproconazole. This lipid-driven retention capacity is independent of the degradation rate constant $k_d$ (“Transport efficiency and degradation”) and is therefore a robust feature of the model regardless of the specific half-life value adopted. This dynamic partitioning has no parallel in static single-compartment models and constitutes a methodological advance for lipophilic pesticides in lipid-accumulating tropical fruits.

### Triazole residues in tropical crops

Cyproconazole is a demethylation-inhibitor (DMI) triazole with $\log K_{OW} = 2.90$. The plant-tissue half-life used in this study (11.5 days nominal, 3.5–16.0-day range; “Transport efficiency and degradation”) is consistent with the fast dissipation generally reported for triazoles in living plant tissue, as opposed to soil. The most directly comparable evidence available is that of Al-Tawieh et al. (2026), who measured dissipation of the structurally related triazole difenoconazole in the fruit of another palm species (date palm, *Phoenix dactylifera* L.) under arid Jordan Valley field conditions across two cultivars (Barhi and Medjool), reporting half-lives of 2.0 and 2.2 days, respectively (k=0.35 and 0.32 day$^{-1}$, $R^2 \ge 0.95$, first-order kinetics, QuEChERS extraction with LC–MS/MS quantification). This is, to our knowledge, the closest available matrix-and-genus analogue to the cyproconazole–coconut endotherapy system modelled here, and it confirms both that triazole half-lives in palm fruit tissue are of the order of days rather than weeks, and that they can be shorter than the 3.5–16.0-day PPDB range used in the present Monte Carlo analysis, which if anything makes the half-life distribution adopted here conservative rather than optimistic. Al-Tawieh et al. (2026) additionally reported significant cultivar-dependent differences in residue persistence within the same palm genus, consistent with the field-variability considerations raised later in this section. Maznah et al. (2015) reported that hexaconazole in oil palm *soils* followed first-order kinetics with an initial rapid phase followed by slower dissipation (a substantially longer process than dissipation from living plant tissue, illustrating the general distinction between soil and plant-tissue dissipation kinetics for triazole fungicides; “Transport efficiency and degradation”). Suganthi et al. (2021) found that tebuconazole—another triazole with similar $\log K_{OW}$—was detected in coconut water, kernel, and leaves at concentrations below the EU MRL of 0.05 μg g$^{-1}$ after field application, suggesting that moderate-lipophilicity triazoles do not accumulate to alarming levels under typical field use rates. The residues reported by Ferreira et al. (2016) for cyproconazole in Brazilian coconut water and pulp were also below EU MRLs, providing empirical support for the general conservatism of the modelling approach used here, even though those field data cannot substitute for a direct experimental validation of this model’s predictions (“Limitations and future work”). The $p_{95}$ concentration from the Monte Carlo analysis for the 1.0 g bimonthly schedule was 0.062–0.068 mg kg$^{-1}$ across fruit ages 5–8 months, consistent with these independent field observations. These field data are therefore best regarded as a supporting comparison, consistent with the general conservatism of the model, rather than as a formal validation of its predictions: neither the application dose nor the treatment history of the sampled palms was controlled or reported, so no quantitative correspondence between measured and predicted residues can be established.

A distinction should be drawn between the two sources of variation involved here. The Monte Carlo analysis quantifies parametric uncertainty only—the propagation of imperfect knowledge of η and $k_d$ through a fixed model structure—whereas residue variability under commercial production additionally reflects cultivar, ambient conditions, tree physiology, and management practice, none of which is represented in the model. The exceedance probabilities in Table 2 should therefore be read as uncertainty in the model’s own prediction for a typical palm, not as the expected dispersion of residues across orchards, which would be wider.

These comparisons, however, all derive from field studies of different pesticide–crop combinations under different agroclimatic conditions, not from coconut endotherapy trials themselves. Actual pre-harvest residues under commercial coconut production will be influenced by factors this model does not represent: ambient temperature and humidity (which govern both vascular transport and enzymatic or photochemical degradation rates), tree age, vascular anatomy and health, irrigation regime, soil type, and the precision of the endotherapy application technique itself. Tropical field conditions in particular—higher and more stable temperatures than the temperate systems from which most triazole dissipation data derive—would be expected to accelerate degradation via the reaction-rate mechanism identified by Fantke et al. (2014) for pesticide dissipation from plants generally, which would work in the direction of shorter effective half-lives and lower residues than modelled here; conversely, periods of water stress or suboptimal tree health could plausibly slow vascular transport and alter η in either direction. Al-Tawieh et al. (2026) provide direct empirical support for both aspects of this argument in a palm fruit system: their arid-climate difenoconazole half-lives (2.0–2.2 days) were markedly shorter than temperate-climate triazole values reported elsewhere (e.g., 3.0–5.5 days in wheat and 3–4 days in grape; Horská et al. 2025), and cultivar identity alone (Barhi vs. Medjool date palm) produced statistically significant differences in residue persistence for every pesticide tested, attributed to differences in fruit morphology, wax composition, and moisture content. Analogous cultivar-level variability in coconut (fruit size, husk thickness, pulp texture across the many Brazilian and international coconut cultivars) is not represented in the present single-parameterisation model and is a plausible additional source of field variation beyond the environmental factors listed above. A further, coconut-specific source of field variability, noted in the “Introduction” section, is cyproconazole’s putative plant-growth-regulator side effect: if endotherapy treatment increases fruit set per bunch as anecdotally observed, and growers respond by manually thinning young fruit, the resulting bunch-level fruit density and the developmental stage at which thinning occurs would both influence per-fruit residue exposure in ways the present single-fruit model does not capture. This interaction between a pesticide’s fungicidal and growth-regulating activity and consequent grower management practice is, to our knowledge, unstudied for cyproconazole in coconut and represents a further argument for field validation (“Limitations and future work”). The PHI recommendations in Tables 1 and 2 should therefore be interpreted as applying to average or typical field conditions within the parameter ranges sampled, not as guarantees under any specific combination of local conditions and management practices.

### Probabilistic risk classification and regulatory implications

The integration of Monte Carlo uncertainty analysis into PHI estimation is increasingly recognised as best practice in pesticide risk assessment (Fantke et al. 2011, 2014). By reporting $P(C_p > \textrm{MRL})$ alongside point estimates, regulators and growers can adopt risk-based rather than purely precautionary decisions. This value is particularly important precisely because several scenarios that appear safe under the deterministic point-estimate model (Table 1) are not safe once parameter uncertainty is propagated (Table 2): for example, the 1.5 g monthly schedule for 5-month fruit has a deterministic peak concentration of 0.091 mg kg$^{-1}$, below the MRL, yet carries a Monte Carlo exceedance probability of 0.249—a risk that the deterministic maximum alone does not communicate. Conversely, for the 1.0 g bimonthly and quarterly schedules, even the $p_{95}$ concentration remains comfortably below the MRL, confirming robust compliance regardless of which of the two model representations is consulted. The absence of crop-specific PHI data for minor-use crops in pesticide registration systems is a well-recognised regulatory gap; the probabilistic harvest-safety matrix developed here offers a practical, model-based tool that can inform discussion of cyproconazole use on coconut under agronomic prescription, subject to the screening-level caveat discussed in the “Limitations and future work” section.

The P<0.05/P<0.20 classification criteria used throughout this study (the “Monte Carlo uncertainty analysis” section) are a model-based risk-communication convention rather than a regulatory limit or a value derived from a specific regulatory standard; Brazilian and Codex frameworks for registered pesticide uses typically require the $p_{95}$ (or an equivalent high percentile) of the residue distribution to remain below the MRL, a criterion this study also reports directly (Table 2 caption; “Monte Carlo analysis: uncertainty propagation”) and which growers or regulators preferring a percentile-based rule can apply without recomputing the model. Because five of the 36 scenarios that are “safe” under P<0.05 would become “moderate risk” under a stricter P<0.01 criterion (the “Monte Carlo uncertainty analysis” section), we recommend that any regulatory use of these results explicitly state which exceedance-probability threshold is being applied, rather than treating the “safe” label as threshold-independent.

### Limitations and future work

This study should be read as a screening-level modelling exercise, not as a validated predictor of field residues. No cyproconazole residue measurements from actual coconut endotherapy trials—with or without this model’s predictions compared against them—are currently available to the authors; the model has been calibrated against a lipid sub-model fit ($R^2=0.94$; “Lipid sub-model and growth calibration”) and against an explicit conservative premise for η (“Transport efficiency and degradation”), neither of which constitutes validation of the final predicted pulp and water concentrations against independent measured residues. Even a single independent field validation point—ideally several, spanning the dose range tested here—would substantially improve confidence in the absolute concentration values reported, as opposed to their relative ranking across scenarios, which is more robust to this limitation since it depends less on the absolute value of η. We recommend that the pre-harvest intervals in Tables 1 and 2 be treated as provisional guidance pending confirmatory residue trials and that any regulatory or commercial use of these results proceed on that basis.

The model assumes that η is dose-independent, so that the proportion of the applied dose reaching a given fruit scales linearly with dose. In practice, endotherapy kinetics may be non-linear at higher doses due to saturation of vascular transport capacity; field trials with multiple dose levels would allow dose-specific calibration of η and would simultaneously resolve the absence-of-validation limitation above. The model also assigns equal degradation rates to pulp and water; future refinements could incorporate compartment-specific half-lives once experimental data become available. The Monte Carlo analysis propagates uncertainty in η and $k_d$ only; the Morris screening (“Global sensitivity analysis”) confirms that the five other parameters tested (partition coefficient, lipid-model and growth-model parameters) contribute negligibly to output uncertainty by comparison, but this screening does not rule out the possibility that other parameters not tested here—notably compartment-specific degradation, or non-linear dose–η relationships—could matter more than assumed. The two propagated parameters, η and $k_d$, were also sampled independently; both are plausibly temperature-dependent and therefore correlated in the field (the “Monte Carlo uncertainty analysis” section), and quantifying this correlation—which would require paired transport and degradation measurements under controlled temperature conditions—is identified as a specific, high-value target for future experimental work. Finally, spatial heterogeneity in endotherapy uptake among individual fruits on the same bunch was not accounted for, nor were the field-level environmental and management factors discussed in the “Triazole residues in tropical crops” section (temperature, tree health, irrigation, application precision), which future field trials should characterise directly rather than infer from the model.

## Conclusions

The Level IV fugacity model coupled with a Gompertz growth function and Monte Carlo uncertainty analysis characterised the risk of cyproconazole residue exceedance in green coconut as a function of dose, application frequency and fruit age at harvest, using the plant-tissue degradation half-life reported for cyproconazole in the Pesticide Properties Database (11.5 days nominal, 3.5–16.0-day range), distinct from the compound’s soil dissipation half-life. A Morris global sensitivity screening across seven model parameters confirmed that transport efficiency (η) and the degradation rate constant ($k_d$) are the dominant sources of output uncertainty, justifying their exclusive propagation in the Monte Carlo analysis, and a multi-seed convergence analysis confirmed that N=2000 realisations were sufficient for stable risk classification. For 1.0 g applied at any of the three tested frequencies, $P(C_p > \textrm{MRL}) \le 0.024$ for all fruit ages (5–8 months), placing this dose in the safe category under the criteria adopted here (the “Monte Carlo uncertainty analysis” section) and under realistic parameter uncertainty. Risk for 1.5 and 2.0 g is age-dependent: younger fruit (5–6 months) carry higher exceedance probabilities than older fruit (7–8 months) because less degradation time has elapsed by harvest. The 1.5 g monthly schedule is not recommended for 5-month fruit (P=0.249) but falls to moderate risk from 6 months onward; the 2.0 g monthly schedule is not recommended at every fruit age tested (P=0.312–0.602), while 2.0 g bimonthly and quarterly schedules are not recommended for young fruit but fall to moderate risk by 7–8 months. Several scenarios classified as deterministically safe (Table 1) are not safe once parameter uncertainty is propagated (Table 2), underscoring that the probabilistic matrix, not the deterministic one, should guide screening-level decisions. The deterministic PHI matrix (Table 1) and the probabilistic harvest-safety matrix (Table 2) together provide a decision-support tool addressing a food-safety gap for a minor-use crop in the absence of officially registered PHI data; however, in the absence of field-residue validation for coconut endotherapy specifically (“Limitations and future work”), these results should be regarded as screening-level guidance, intended to prioritise experimental confirmation and to inform interim agronomic practice, rather than as a substitute for supervised residue trials before formal regulatory adoption. The modelling framework itself—Level IV fugacity coupled with dynamic growth functions, Monte Carlo uncertainty propagation and Morris sensitivity screening—is transferable to other systemic fungicides applied via endotherapy to tropical palms, provided the same caveats regarding half-life sourcing and field validation are observed.

## Data Availability

All data generated during this study are included in the present article (Tables 1 and 2 and equations in the “Materials and methods” section) and in the supplementary material (full concentration matrices, probabilistic harvest-safety matrix, and commented Python 3.12 source code). No external data repositories were used.

## Declarations

### Use of artificial intelligence

Generative artificial intelligence tools (Claude, Anthropic; DeepSeek) were used extensively during the preparation and revision of this manuscript. This assistance included the following: drafting and revising text across all sections, including in response to editor and reviewer comments; writing, executing, and debugging the Python 3.12 code implementing the Level IV fugacity model, the Monte Carlo uncertainty analysis, the Morris global sensitivity screening, and the multi-seed convergence analysis reported in the “Materials and methods”–“Results” sections; generating Fig. 1 (composite concentration-curve panel) from the underlying model output; and verifying, correcting, and reformatting the reference list, including identifying and correcting citation errors (incorrect authors, years, volumes, pages, and DOIs) present in earlier drafts of this manuscript. The author directed this work throughout, specified all scientific questions and modelling decisions, and reviewed, validated, and takes full responsibility for all code, numerical results, text, and conclusions in the final manuscript. No AI tool is listed as an author or made independent scientific judgments, hypotheses, or interpretive decisions without the author’s review and approval.
